# Mechanisms of Stress-Induced Spermatogenesis Impairment in Male Rats Following Unpredictable Chronic Mild Stress (uCMS)

**DOI:** 10.3390/ijms20184470

**Published:** 2019-09-10

**Authors:** Peng Zou, Xiaogang Wang, Wang Yang, Chang Liu, Qing Chen, Huan Yang, Niya Zhou, Yingfei Zeng, Hongqiang Chen, Guowei Zhang, Jinyi Liu, Jia Cao, Lin Ao, Lei Sun

**Affiliations:** 1 Key Lab of Medical Protection for Electromagnetic Radiation, Ministry of Education of China, Institute of Toxicology, College of Preventive Medicine, Third Military Medical University, Chongqing 400038, China; 2 Frontier Defence Medical Service Training Group, Third Military Medical University, Hutubi 831200, China; 3 Department of Environmental Health, College of Preventive Medicine, Third Military Medical University, Chongqing 400038, China

**Keywords:** uCMS, GR, spermatogonia, spermatids, apoptosis, G_0_/G_1_ cell cycle arrest

## Abstract

The negative association between psychological stress and male fertility has been known for many years. This study was aimed at (i) identifying spermatogenesis impairment induced by psychological stress in rats and (ii) exploring the role of glucocorticoid receptor (GR) signaling in these adverse effects (if they exist). Male Sprague Dawley rats were exposed to a six-week period of unpredictable chronic mild stress (uCMS) along with cotreatment of GR antagonist RU486 (1 mg/kg/day). Testicular damage was assessed by testicular pathological evaluation, epididymal sperm concentration, serum testosterone levels, testicular apoptotic cell measurements, and cell cycle progression analyses. Rats in the uCMS group had decreased levels of serum testosterone and decreased epididymal sperm concentration. The uCMS-treated rats also had decreased numbers of spermatids and increased levels of apoptotic seminiferous tubules; additionally, cell cycle progression of spermatogonia was arrested at the G_0_/G_1_ phase. Furthermore, uCMS exposure caused an increase in serum corticosterone level and activated GR signaling in the testes including upregulated GR expression. RU486 treatment suppressed GR signaling and alleviated the damaging effects of stress, resulting in an increased epididymal sperm concentration. Overall, this work demonstrated for the first time that the activation of GR signaling mediates stress-induced spermatogenesis impairment and that this outcome is related to cell apoptosis and cell cycle arrest in germ cells.

## 1. Introduction

The negative association between psychological stress and male fertility has been known for many years. The first published study that examined the effect of psychological stress on testicular spermatogenesis was conducted in 1977 [1]. These researchers found that Sertoli cells and spermatogonia were the only cell types found in the seminiferous tubules in some prisoners who were kept waiting for a long time before execution. In recent years, epidemiological studies found that there is a negative relationship between various psychological stress factors and male fertility [2,3,4]. In a study of 1215 young Danish men, researchers found that subjects with high levels of self-reported stress had worse semen parameters than subjects with low levels of self-reported stress [2]. This phenomenon has also been demonstrated in animal experiments. For example, immobilization stress induced downregulation of testosterone synthesis genes such as scavenger receptor class b member 1 (*Scarb1*), steroidogenic acute regulatory gene (*Star*), and cytochrome P450 family 17 subfamily A member 1 (*Cyp17a1*) expression in testes; this downregulation led to a significant decrease in plasma testosterone levels [5]. Using a cold water immersion-induced stress model in rats, Juarez-Rojas et al. reported that stress can potentially activate intrinsic/extrinsic apoptosis pathways in testes resulting in reduced testosterone levels [6]. Glucocorticoids, which are a hallmark of stress, were found to have similar toxic effects on male reproduction. Researchers found that dexamethasone-treated rats had 10 times more apoptotic germ cells than the control group [7].

Glucocorticoid receptor (GR)-mediated apoptosis and the inhibition of testosterone synthesis in Leydig cells are considered the main mechanisms of stress-induced spermatogenesis impairment [8]. For example, Orr et al. reported that immobilization stress resulted in increased levels of plasma glucocorticoids in male rats and that the decline of testosterone production in male rats following immobilization stress was in part mediated by a direct action of glucocorticoids which was mediated via GRs on Leydig cells [9]. However, there are still many unresolved issues that deserve further study. For example, since GRs are also expressed in spermatogonia and spermatocytes that are not direct targets of testosterone [10,11], whether stress-induced spermatogenesis impairment is mediated by the direct action of glucocorticoids via GRs on spermatogonia and spermatocytes is not clear and deserves further study. Additionally, previous studies that used a single predictable stress, such as immobilization stress [5,9,12], cold water immersion [6], or direct exposure to exogenous glucocorticoids [7,13], instead of a chronic unpredictable stress may not present actual stress faced by humans or animals in real life.

Based on this background information, it is clear that the mechanisms that mediate the effects of chronic stress on male reproduction, especially in germ cells, are not fully understood. The objective of our study was to identify spermatogenesis impairment induced by psychological stress in rats, and if adverse effects were identified, the study aimed to conduct additional tests to establish whether cotreatment of a GR antagonist (RU486) could alleviate these damaging effects. The present study used the unpredictable chronic mild stress (uCMS) rat model, which is a commonly used, reliable, and effective model that best simulates chronic psychological stress in humans [14]. Our data revealed that uCMS-treated rats had increased levels of apoptotic seminiferous tubules; additionally, the cell cycle progression of spermatogonia was arrested at the G_0_/G_1_ phase. RU486 treatment suppressed GR signaling and alleviated the damaging effects of stress, resulting in an increased concentration of epididymal sperm. Collectively, this work demonstrated for the first time that the activation of GR signaling mediates stress-induced spermatogenesis impairment and that this outcome is related to cell apoptosis and cell cycle arrest in germ cells.

## 2. Results

### 2.1. The Procedure and Verification of the uCMS Model

**Figure 1:**
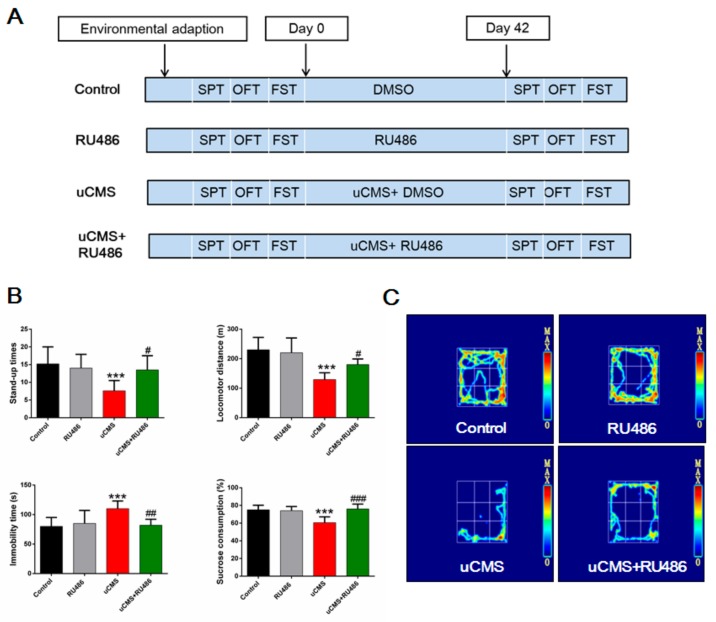
Study design and verification of the uCMS model. (**A**) Schematic diagram of the study design. (**B**) Results of behavioral tests including OFT, FST, and SPT (*n* = 10). (**C**) Heat map of the rats’ motion trail in the OFT. Data were analyzed by one-way ANOVA with post hoc multiple comparisons test. *p < 0.05, **p < 0.01, and ***p < 0.001 compared with the control group; #*p* < 0.05, ##*p* < 0.01, and ###*p* < 0.001 compared with the uCMS group. uCMS = unpredictable chronic mild stress; ANOVA = analysis of variance; OFT = open field test; FST = forced swimming test; SPT = sucrose preference test.

The study design and uCMS procedure are presented in Figure 1A and Supplementary Table S1, respectively. Following six weeks of uCMS exposure (Figure 1B), rats in the uCMS group (stress + vehicle) showed decreased stand-up times, locomotor distance, and sucrose consumption (%), and increased immobility time (all *p* values < 0.001) as compared to the control group (no stress + vehicle). However, rats in the uCMS + RU486 group showed increased stand-up times (*p* = 0.010), locomotor distance (*p* = 0.015), sucrose consumption (%) (*p* < 0.001), and decreased immobility time (*p* = 0.002) as compared to rats in the uCMS group (stress + vehicle). These results demonstrate that the uCMS model was successfully established and that RU486 was associated with antistress effects. Figure 1C shows a heat map of the rats’ motion trail in the open-field test (OFT).

### 2.2. Effects of Stress on Body Weight, Testicular Structure, and Semen Parameters

**Figure 2:**
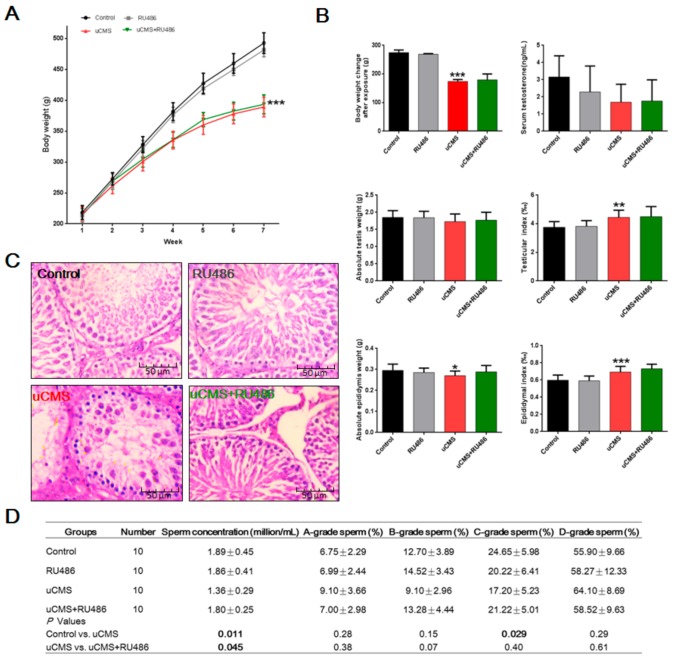
Effects of stress on body weight, testicular structure, and semen parameters. (**A**) Body weight measured during uCMS exposure (*n* = 10). (**B**) Body weight change, serum testosterone levels, absolute testis and epididymis weights, and testicular or epididymal index after uCMS exposure (*n* = 10). Testis (or epididymis) index = absolute testis (or epididymis) weight/body weight. (**C**) Histological changes in testicular structure (400× magnification). (**D**) Sperm concentration and motility were assessed by the CASA System (*n* = 10). Sperm motility has four grades: (i) rapid forward and linear moving (a-grade), (ii) slowly or sluggishly moving (b-grade), (iii) in situ but not forward moving (c-grade), and (iv) not moving (d-grade). A repeated-measures two-way ANOVA was used to determine differences in weight change over time, and the rest of the data were analyzed using one-way ANOVA with post hoc multiple comparisons test. *p < 0.05, **p < 0.01, and *** *p* < 0.001 compared with the control group; ^#^*p* < 0.05, ^##^*p* < 0.01, and ^###^*p* < 0.001 compared with the uCMS group. uCMS = unpredictable chronic mild stress; ANOVA = analysis of variance; CASA = computer-aided sperm analysis.

As shown in Figure 2A, a repeated-measures two-way ANOVA of body weight revealed significant differences in between-subject variation (F (3,140) = 148.3, *p* < 0.001), within-subject variation (F (6,140) = 908.2, *p* < 0.001), and interaction of group × time (F (18,140) = 14.49, *p* < 0.001). After six weeks of uCMS treatment (Figure 2B), rats in the uCMS group had significant decreased body weight change after exposure (173.50 ± 7.23 versus 274.67 ± 8.91 g, *p* < 0.001) and absolute epididymis weight (0.27 ± 0.02 versus 0.30 ± 0.03 g, *p* = 0.041) as compared to control group rats. There was no statistically significant difference in testis weight (1.73 ± 0.22 versus 1.85 ± 0.20 g, *p* = 0.58) and serum testosterone levels (1.69 ± 1.04 versus 3.16 ± 1.23 ng/mL, *p* = 0.06) between the uCMS group and the control group. However, testicular index (4.44 ± 0.41 versus 3.75 ± 0.39‰, *p* < 0.01) and epididymal index (0.69 ± 0.06 versus 0.60 ± 0.06‰, *p* < 0.001) were increased in uCMS-treated rats as compared to rats in the control group. We also observed significant testicular structural damage in rats following chronic stress exposure. H&E-stained sections of testes showed that significant histological changes occurred in seminiferous tubules (Figure 2C). uCMS-treated rats showed decreases in seminiferous tubule diameter (135.12 ± 4.80 versus 210.79 ± 5.22 μm; *p* < 0.001) and epithelial height (50.38 ± 4.91 versus 75.93 ± 4.79 μm, *p* < 0.001) as compared to control group rats. Additionally, significant decreases in sperm concentration (1.36 ± 0.29 versus 1.89 ± 0.45 million/mL, *p* = 0.011) and C-grade sperm (%) (17.20 ± 5.23 versus 24.65 ± 5.98%, *p* = 0.029) were detected in rats exposed to chronic stress (Figure 2D). Our data also demonstrated that RU486 cotreatment alleviated the toxic testicular-related effects of chronic stress and improved testicular structure. Rats in the uCMS + RU486 groups had an enlarged seminiferous tubule diameter (200.47 ± 4.37 versus 135.12 ± 4.80 μm, *p* < 0.001), increased epithelial height (71.00 ± 4.30 versus 50.38 ± 4.91 μm, *p* < 0.001), and higher sperm concentration (1.80 ± 0.25 versus 1.36 ± 0.29 million/mL, *p* = 0.045) as compared to the rats in the uCMS group. When compared to rats in the control group, rats in the uCMS + RU486 groups had a decreased seminiferous tubule diameter (200.47 ± 4.37 versus 210.79 ± 5.22 μm; *p* < 0.001). RU486 administration did not ameliorate the decline in weight gain (*p* = 0.83) or serum testosterone (*p* = 0.90) in rats exposed to chronic stress.

### 2.3. Stress Decreases the Number of Spermatids

**Figure 3:**
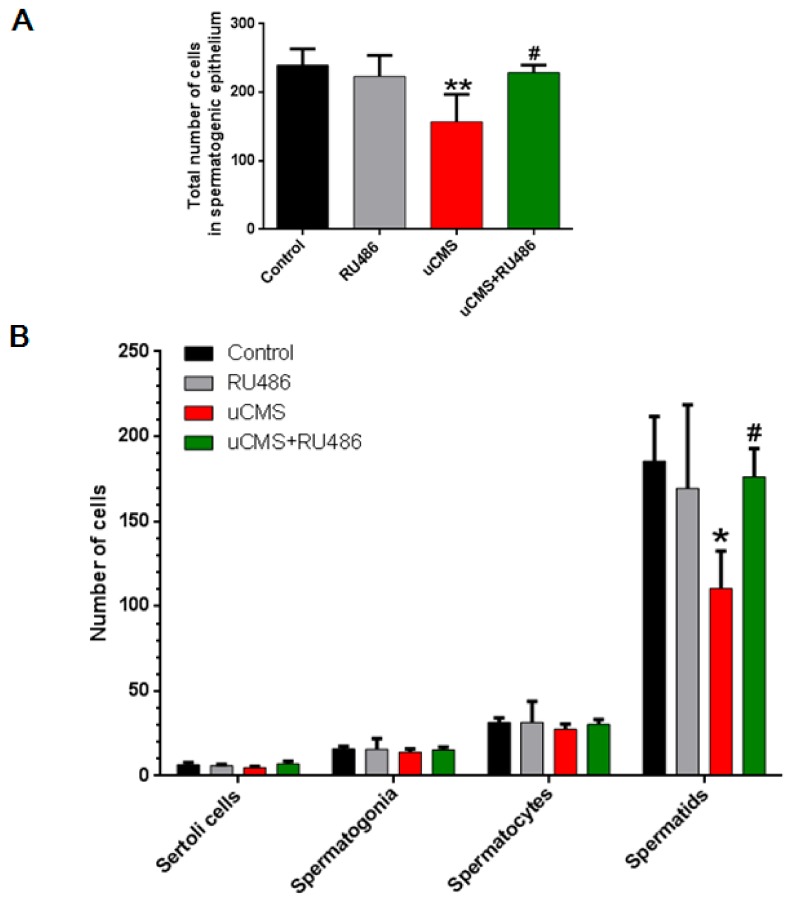
Stress decreased the number of spermatids. (**A**) Total number of cells in the spermatogenic epithelium. (**B**) The number of Sertoli cells, spermatogonia, spermatocytes, and spermatids in the spermatogenic epithelium. Three tubules per animal in each group were randomly selected and analyzed (*n* = 10). Data were analyzed by one-way ANOVA with post hoc multiple comparisons test. *p < 0.05, **p < 0.01, and *** *p* < 0.001 compared with the control group; ^#^*p* < 0.05 compared with the uCMS group. ANOVA = analysis of variance; uCMS = unpredictable chronic mild stress.

The aforementioned results show that the seminiferous tubules of the uCMS group underwent significant pathological changes. To further explore the cells targeted by stress-induced spermatogenesis impairment, cells were counted within the spermatogenic epithelium (including Sertoli cells, spermatogonia, spermatocytes, and spermatids). The different types of cells within the spermatogenic epithelium are marked in Supplementary Figure S1A, and refer to the description presented in a previous study [15], and the spermatogenic epithelium of SD rats in different groups is shown in Supplementary Figure S1B. As shown in Figure 3A,B, the total number of cells within the spermatogenic epithelium decreased significantly in the uCMS group as compared to the control group (156.75 ± 40.44 versus 239.50 ± 23.81, *p* = 0.007). This decrease may have contributed to the marked decrease in the number of spermatids in the uCMS group (110.50 ± 21.98 versus 185.50 ± 26.36, *p* = 0.023). In addition, RU486 cotreatment caused a significant increase in the total number of cells within the spermatogenic epithelium (228.75 ± 10.87 versus 156.75 ± 40.44, *p* = 0.018) and spermatids numbers (176.25 ± 16.64 versus 110.50 ± 21.98, *p* = 0.048). These data indicate that the chronic stress-induced decrease in sperm concentration may originate from a decrease in the number of germ cells in the seminiferous tubules, especially from the specific reduction of spermatids.

### 2.4. Stress Induces Apoptosis of Spermatids

**Figure 4:**
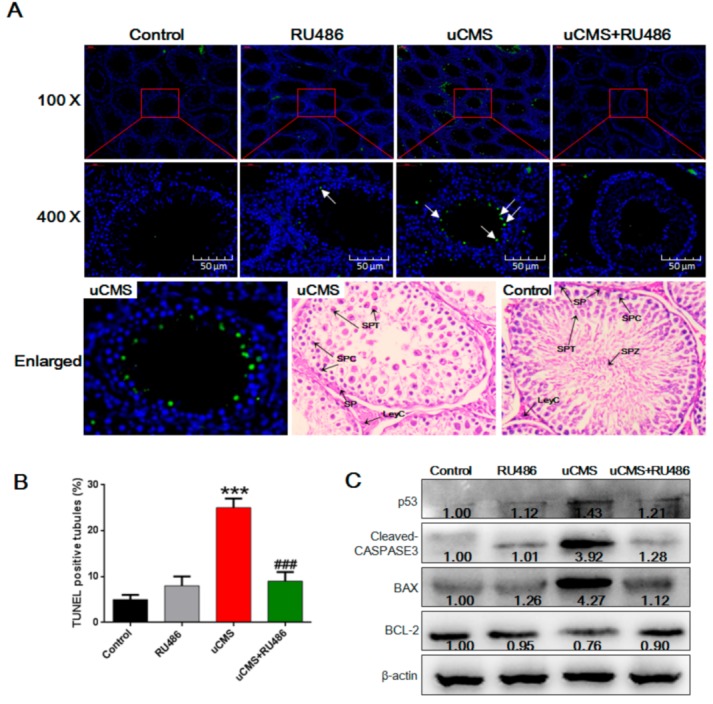
Stress induces apoptosis in spermatids and the apoptotic cascade in testes. (**A**) Testicular cell apoptosis was detected by TUNEL staining. The white arrow indicates apoptotic cells (most were spermatids) within seminiferous tubules. To clarify which stage of germ cells was TUNEL-positive, an enlarged TUNEL-stained image from the uCMS group and the image stained with H&E from the control and the uCMS group are shown. (**B**) Percentages of seminiferous tubules with more than six TUNEL-positive cells (*n* = 10). (**C**) Protein levels of p53, BAX, BCL-2, and cleaved CASPASE 3 were detected by Western blot analysis (*n* = 10). Data were analyzed by one-way ANOVA with post hoc multiple comparisons test. *p < 0.05, **p < 0.01, and ***p < 0.001 compared with the control group; ^#^*p* < 0.05, ^##^*p* < 0.01, and ^###^*p* < 0.001 compared with the uCMS group. TUNEL = terminal deoxynucleotidyl transferase-mediated dUTP-biotin nick end labeling; SP = spermatogonia; SPC = spermatocytes; SPT = spermatids; SPZ = spermatozoa; LeyC = Leydig cell; BCL-2 = B cell leukemia/lymphoma 2; BAX = BCL-2-associated X; ANOVA = analysis of variance.

To further elucidate the reasons for the reduction in spermatids, apoptosis of testicular cells was investigated using the terminal deoxynucleotidyl transferase-mediated dUTP-biotin nick end labeling (TUNEL) method. According to the analytical methods of a previous study [16], we found that the proportion of TUNEL-positive tubules (tubules with more than six TUNEL-positive cells) was significantly increased in the uCMS group (Figure 4A,B, 25.02 ± 2.11% versus 5.22 ± 1.00%, *p* < 0.001). Based on microscopy (400), the majority of apoptotic cells were spermatids. These results indicate that chronic stress may reduce the number of spermatids by inducing cell apoptosis. The levels of proteins associated with the apoptotic pathway were analyzed (Figure 4C). Compared to the controls, the activity of cleaved CASPASE 3, p53, and the ratio of the BCL-2-associated X (BAX; a proapoptotic protein) to B cell leukemia/lymphoma 2 (BCL-2; an antiapoptotic protein) were significantly increased (all *p* values < 0.001). RU486 cotreatment significantly ameliorated testicular cell apoptosis induced by uCMS (9.13 ± 2.36% versus 25.02 ± 2.11%, *p* < 0.001) and caused a marked normalization of protein levels in the apoptotic pathway (all *p* values < 0.05).

### 2.5. Stress Induces Cell Cycle Arrest at the G_0_/G_1_ Phase in Spermatogonia

**Figure 5:**
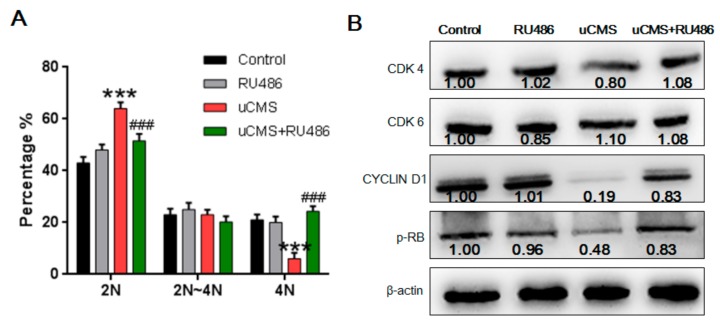
Stress induces cell cycle arrest at the G_0_/G_1_ phase in spermatogonia. (**A**) DNA content distribution of early spermatogenic cells including spermatogonia (DNA content: 2N~4N) and primary spermatocytes (DNA content: 4N) was assessed using the propidium iodide method along with flow cytometry (*n* = 10). (**B**) G_0_/G_1_ phase-related proteins were detected by Western blotting analysis (*n* = 10). Data were analyzed by one-way ANOVA with post hoc multiple comparisons test. * *p* < 0.05, ** *p* < 0.01, and *** *p* < 0.001 compared with the control group; ^#^*p* < 0.05, ^##^*p* < 0.01, and ^###^*p* < 0.001 compared with the uCMS group. ANOVA = analysis of variance; uCMS = chronic unpredictable mild stress; CDK4 = Cyclin-dependent kinase 4; CDK6 = Cyclin-dependent kinase 6; p-RB = phospho-Retinoblastoma.

Figure 5 shows the distribution of DNA content of early spermatogenic cells, including spermatogonia (DNA content: 2N~4N) and primary spermatocytes (DNA content: 4N). The “N” in our study represents DNA content, not the number of chromosomes. 4N germ cells include primary spermatocytes and G_2_-phase spermatogonia in the mitotic cell cycle [17]. As shown in Figure 5A, uCMS treatment significantly increased the proportion of 2N cells (63.80 ± 2.71% versus 43.30 ± 3.10%, *p* < 0.001). There were no significant differences in the number of spermatocytes between each group, according to the results shown in Figure 3B (*p* = 0.81). Thus, a reduction in 4N germ cells may be due to the decrease in G_2_-phase spermatogonia (4N) but not to the decrease in spermatocyte numbers. In other words, the increased proportion of 2N cells may be due to mitotic cell cycle arrest of spermatogonia at the G_0_/G_1_ phase. The results of the Western blot analysis further support this hypothesis (Figure 5B). Levels of three important regulatory proteins (cyclin-dependent kinase 4 [CDK4], CYCLIN D1, and phospho-Retinoblastoma [*p*-RB]) that are necessary for transformation of G_0_/G_1_ phase to S phase were significantly decreased (*p* = 0.030, *p* < 0.001, and *p* < 0.001, respectively). Similarly, RU486 cotreatment normalized the proportion of 2N cells (50.15 ± 2.91% versus 63.80 ± 2.71%, *p* < 0.001) and caused an increase in the protein levels of CDK4, CYCLIN D1, and *p*-RB (*p* = 0.006, *p* < 0.001, and *p* = 0.016, respectively). The above results indicate that chronic stress induces cell cycle arrest of spermatogonia at the G_0_/G_1_ phase.

### 2.6. Stress Activates the Hypothalamic-Pituitary-Adrenal (HPA) Axis and Elevates GR Levels in Testes

**Figure 6:**
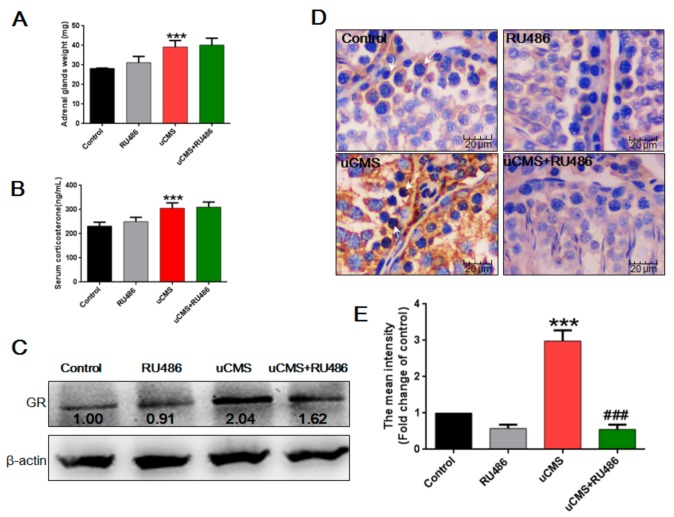
Stress activated the HPA axis and elevated GR levels in the testes. (**A**) uCMS exposure increased the weight of the adrenal glands (*n* = 10). (**B**) uCMS exposure increased the serum corticosterone level (*n* = 10). (**C**) The protein levels of GR in testes were detected by Western blotting (*n* = 10). (**D**) Immunohistochemistry was used to detect the in situ protein levels of GR in testes (*n* = 10,400× magnification). White arrows indicated the GR-positive cells in testes. (E) Immunoreactivity intensity of GR was analyzed using Image Pro Plus6.0 software. Data were analyzed by one-way ANOVA with post hoc multiple comparisons test. * *p* < 0.05, ** *p* < 0.01, and *** *p* < 0.001 compared with the control group; ^#^*p* < 0.05, ^##^*p* < 0.01, and ^###^*p* < 0.001 compared with the uCMS group. HPA = Hypothalamic–pituitary–adrenal; GR = glucocorticoid receptor; uCMS = unpredictable chronic mild stress; ANOVA = analysis of variance.

Adrenal gland weight (39.19 ± 3.33 versus 28.29 ± 3.11 mg, *p* < 0.001) and serum corticosterone levels (305.47 ± 22.00 versus 231.92 ± 15.73 ng/mL, *p* < 0.001) were higher in stressed rats as compared to the control group (Figure 6A,B). In addition, Western blot analysis (Figure 6C) showed that stress induced an average twofold increase in GR protein expression level in the testes, as compared to the control (*p* < 0.001). Immunohistochemistry was used to detect the in situ expression of GRs in the testes. As shown in Figure 6D, control rats showed a low basal expression level of GR, while rats in the uCMS group showed a strong positive staining of GR expression in male reproductive cells, especially in spermatogonia and spermatocytes. Quantitative analysis (Figure 6E) showed that uCMS exposure increased GR expression approximately threefold as compared to control rats (*p* < 0.001). With respect to the effects of RU486 on uCMS-induced GR expression, results of Western blot and immunohistochemistry showed that RU486 partially reversed the increased levels of GR protein induced by uCMS exposure (Figure 6C,D, *p* < 0.001). However, no significant differences were found between the uCMS and uCMS + RU486 groups for the adrenal gland weight (39.19 ± 3.33 versus 40.20 ± 3.51 mg, *p* > 0.05) and levels of serum corticosterone (305.47 ± 22.00 versus 310.01 ± 21.02 ng/mL, *p* > 0.05) (Figure 6A,B). These data indicate that stress activates the HPA axis, resulting in elevated adrenal gland weight and serum corticosterone levels, while the GR antagonist RU486 had no effect on the activation of the HPA axis by uCMS but alleviated high GR levels induced by uCMS in the testes.

## 3. Discussion

The decline in male semen quality, first reported by Carlsen et al. in 1992 [18], is an indisputable fact. The effects of psychological stress on male reproductive health is an interesting and worthwhile area of study. Our previous studies demonstrated that depression and work-related stress were negatively associated with sperm concentration and total sperm count in Chinese men [19,20], which is consistent with findings from other countries [2,4]. In this study, spermatogenesis impairment was induced by chronic stress in the uCMS rat model. Exposure to chronic stress over a six-week period significantly induced cell cycle arrest of spermatogonia at the G_0_/G_1_ phase and increased the number of apoptotic spermatids. RU486 cotreatment during stress markedly normalized the cell cycle progression of spermatogonia and almost completely alleviated the apoptosis of spermatids, accompanied by GR downregulation. These results demonstrate for the first time that spermatogenesis impairment induced by chronic stress is associated with apoptosis and cell cycle arrest of germ cells, which may be due to the activation of GR signaling.

**Figure 7:**
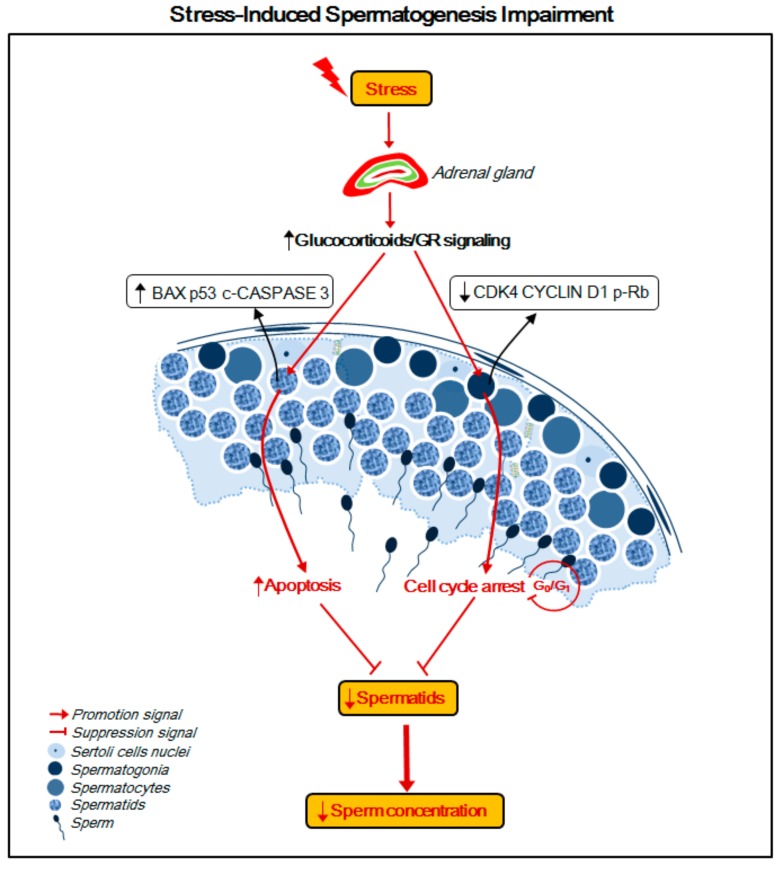
Schematic diagram of the proposed model for stress-induced spermatogenesis impairment. A proposed molecular mechanism for the stress-induced decrease of sperm concentration in the rat epididymis involves two parts: (i) stress activates GR signaling due to increased levels of glucocorticoids, which then induces spermatids apoptosis via upregulating BAX, p53, and cleaved CASPASE 3 and (ii) stress inhibits cell cycle progression of spermatogonia at the G_0_/G_1_ phase by downregulation of CDK4, CYCLIN D1, and p-RB. Both of these two aspects may explain the decreased number of spermatids, leading to reduced epididymal sperm concentration.

Our data suggest that the molecular mechanism of stress-induced spermatogenesis impairment involves two parts (summarized in Figure 7). First, chronic stress induces cell cycle arrest in spermatogonia at the G_0_/G_1_ phase. Spermatogonia, the diploid progenitor of all germ cell types, have the dual responsibility of maintaining meiosis during male gamete production and undergoing mitosis [21]. Thus, an inhibition of cell cycle progression of spermatogonia may lead to a decrease in the number of spermatids and mature sperm cells that differentiate from spermatogonia. Second, the TUNEL results showed that the majority of the stress-induced apoptotic testicular cells were spermatids, which led to a reduction in the number of spermatids and luminal sperm per seminiferous tubule. Based on these two sets of information, a stress-induced decrease in sperm concentration may be caused by cell cycle arrest of spermatogonia and increased apoptosis of spermatids. Stress or glucocorticoid-induced germ cell apoptosis was detected in several previous studies. The results of our study demonstrated that G_0_/G_1_ phase cell cycle arrest in spermatogonia may be another mechanism of stress-induced spermatogenesis impairment. Treatment with RU486 may normalize cell cycle progression in spermatogonia. These results suggest that GR signaling may mediate stress-induced G_0_/G_1_ phase cell cycle arrest in spermatogonia, which may represent a possible drug intervention target.

The keys to understanding the effects of stress on male reproduction are the sympathetic–adrenal system (SAS) and the hypothalamic–pituitary–adrenal (HPA) axis, which are essential for acute and chronic stress responses, respectively [22]. Acute stress can induce an individual′s adaptive response to the environment. However, chronic, excessive stress causes cumulative adverse impacts on health outcomes [23]. Under conditions of chronic stress, the paraventricular nucleus (PVN) of the hypothalamus senses external stimuli and activates the secretion of corticotropin-releasing and adrenocorticotropic hormones (CRH and ACTH, respectively) [24]. Chronic stress activates the HPA axis and suppresses hypothalamic–pituitary–gonadal (HPG) function via increased glucocorticoids at all levels. For example, chronic stress suppresses the release of gonadotropin-releasing hormone (GnRH), which is thought to be mediated by the GRs, leading to decreased secretion of luteinizing and follicle-stimulating hormones (LH and FSH, respectively) [25]. Chronic stress could also inhibit testosterone synthesis and apoptosis of Leydig cells, leading to dysfunction of the receptor G-protein-coupled receptor (GPR54) system, and damage the blood–testis barrier (BTB) [10,26,27,28]. The mechanism for modulating glucocorticoid control of testosterone biosynthesis in the Leydig cells has been clearly described in a review [29].

In the present study, we report that uCMS induced apoptosis of seminiferous tubules and increased the protein levels of cleaved CASPASE 3, p53, and BAX in testes. These results were similar to the results of previous studies. For example, Sun et al. reported that restraint stress induced testicular cell apoptosis, activation of the apoptotic cascade in testes, and increased levels of serum corticosterone [16]. These damaging effects of restraint stress could be mitigated by melatonin, one of the most well-investigated antioxidants. These results indicate that oxidative stress may be a possible explanation for stress-induced testicular cell apoptosis. Another study that used dexamethasone, a synthetic glucocorticoid, drew similar conclusions [13]. They found that the apoptotic index of germ cells was significantly increased in rats treated with 7 or 10 mg·kg^−1^ of dexamethasone for seven days. Yazawa et al. further demonstrated that glucocorticoid-induced germ cell apoptosis was mediated by GRs [7]. Correctively, the results from our study and previous studies indicate that testicular cells are susceptible to stress-induced cell apoptosis mediated by increased levels of glucocorticoids and GR signaling. One issue that should be noted is that the most frequently used models in previous studies were immobilization stress [5,9,12], cold water immersion [6], and direct exposure to exogenous glucocorticoids [7,13]. These stressors represent a single predictable stress that does not reflect actual stress faced by humans and animals in real life. When animals are exposed to acute and severe stress, both the sympathetic–adrenal system (SAS) and the hypothalamic–pituitary–adrenal (HPA) axis are activated, which increases tension in the nervous system and stimulates gluconeogenesis to provide energy for the “flight or fight” response [22,30]. In conditions of chronic mild stress, the HPA axis, not the SAS, is regarded as the main feature of stress [23]. Thus, uCMS is a more reliable animal model for examining the effect of stress and the role of glucocorticoids. Furthermore, since glucocorticoids can act on a plethora of tissues throughout the body to regulate a variety of essential biological functions such as cell metabolism [31], cell apoptosis [32], and neurodevelopment [33], conclusions from studies that used exogenous administration of glucocorticoids should be confirmed to verify the role of glucocorticoids and GR signaling in chronic stress-induced spermatogenesis impairment. Our study used the uCMS model, which is a commonly used, reliable, and effective model that best simulates chronic psychological stress in humans, to demonstrate the role of glucocorticoids and GR signaling in chronic stress-induced spermatogenesis impairment.

Although GRs were reported to be expressed in spermatogonia, spermatocytes, Leydig and Sertoli cells, macrophages, fibroblasts, smooth muscle cells, and endothelial cells of blood vessels in the testes [11,34,35], studies on the function of GRs expressed by these cells are limited. Only one study, which used the Sertoli cell GR knockout model in mice [11], reported that the GRs in Sertoli cells were required for maintaining normal numbers of Sertoli/germ cells. Whether the GR, especially in spermatogonia, is involved in stress-induced spermatogenesis impairment is still unknown. In the present study, we found that RU486 had no effect on uCMS-activated HPA axis but could partially inhibit uCMS-induced high GR expression levels in testes. This finding may be explained by the fact that the HPA axis is initially activated by stress, which is mediated by the PVN of the hypothalamus [24]. The activated HPA axis induces an increase in the synthesis and secretion of corticosterone by the adrenal cortex, resulting in high corticosterone levels in serum. On the other hand, RU486, an antiglucocorticosteroid agent, suppresses GR signaling and could alleviate only some of the adverse effects induced by high glucocorticoid levels via inhibition of glucocorticoid binding to its receptors [36]. In the present study, RU486 suppressed GR signaling and inhibited uCMS-induced GR expression in testes, but had no significant effects on activation of the HPA axis. GR expression in spermatogonia and spermatocytes was significantly upregulated after six weeks of chronic stress exposure, as a result of HPA axis activation. RU486 cotreatment significantly reduced the expression of abnormally activated GRs in spermatogonia and reduced the degree of stress-induced testicular damage. Therefore, chronic stress-induced germ cell apoptosis and G_0_/G_1_ cell cycle arrest of spermatogonia are most likely to be directly mediated by the GR. Results from previous studies support our deduction. First, previous studies have demonstrated that the most important target cells of testosterone are Sertoli cells, not spermatogonia [10]. Secondly, many studies reported that glucocorticoids inhibit cell proliferation by arresting the cell cycle at the G_0_/G_1_ phase [37,38], which is similar to the results of our study. For example, Goya et al. reported that glucocorticoids induced a specific block of cell cycle progression at G_0_/G_1_ phase in mammary cells [38]. To the best of our knowledge, our study is the first to demonstrate stress-induced G_0_/G_1_ phase cell cycle arrest in spermatogonia.

Our study revealed that uCMS treatment had no (or little) effect on absolute testis weight (or absolute epididymis weight) but may have caused a decrease in the body weight of rats. The reasons may be that stress-induced increased levels of glucocorticoids can cause metabolic imbalance [39], which possibly leads to the decreased body weight in stressed rats. Another reason may be that unlike most previous studies that used a single or acute stimulus (e.g., immobilization stress and cold water immersion) with high intensity, the eight stimuli in uCMS were of moderate intensity and therefore may not have induced a significant reduction in the weight of testis. Our study found that RU486 cotreatment did not completely abrogate stress-induced spermatogenesis impairment (e.g., Figure 4B shows that the proportion of TUNEL-positive tubules in uCMS + RU486 group was still higher than the control group); thus, we cannot rule out the effect of other factors, such as testosterone. Testosterone is one of the most important regulatory factors of spermatogenesis [40]. Although previous studies reported that GR signaling is involved in glucocorticoid-induced inhibition of testosterone synthesis [41], we did not observe significant rescue of decreased testosterone levels by RU486 in the condition of uCMS exposure. Reasons for this may be that, besides GR signaling, many other factors, pathways, and molecular mechanisms may participate in stress-induced inhibition of testosterone biosynthesis, such as the serotonergic system [42], GnRH [43], and ghrelin [44]. In future studies, we will further explore the role of testosterone in spermatogenesis under conditions of uCMS exposure. In addition, studies should be conducted using germ cell lines treated with glucocorticoids in vitro or germ cell GR knockout animal models. Our study identified the effects of chronic stress on different types of germ cells and provides clues for future studies aimed at further examination of the function of GRs expressed by spermatogonia and spermatocytes.

In conclusion, using the uCMS model, we found that six weeks of chronic stress exposure induced cell cycle arrest of spermatogonia at the G_0_/G_1_ phase and caused an increase in apoptotic spermatids, possibly leading to the decreased number of spermatids, which may be due to the activation of GR signaling in testes. RU486 cotreatment facilitated a significant normalization of GR expression in the testes and ameliorated stress-induced spermatogenesis impairment. Our study offers new insight into the decline of semen quality caused by psychological stress in humans and provides clues for future studies to examine the function of GRs expressed by spermatogonia and spermatocytes.

## 4. Materials and Methods

### 4.1. Animals

Forty adult male Sprague Dawley rats (weight 140–160 g) were obtained from the Experimental Animal Center, Institute of Surgery, Daping Hospital, Third Military Medical University, in Chongqing, China. Upon arrival, the rats were housed in a controlled environment (a 12 h light/dark cycle with lights on at 08:00, 25 ± 1 °C, 50–60% humidity, and free access to water and food) and were allowed to adapt to the environment for seven days before initiating the stress procedures. All data were collected between September 2016 and February 2018.

### 4.2. Ethics Statement

Protocols were conducted in accordance with the Guide for Care and Use of Laboratory Animals and were approved by the Institutional Committee of Laboratory Animal Experimentation at the Third Military Medical University in China (License number: SYXK-PLA-20120056, 9 June 2016).

### 4.3. Drug Solutions and Administrations

RU486 (mifepristone) was purchased from Sigma (St. Louis, MO, USA) and was dissolved in vehicle (dimethyl sulfoxide [DMSO]) at a concentration of 2 mg/mL. Rats were treated 1 h before each stressful activity with vehicle or RU486 via subcutaneous injection at a volume of 1 mg/kg/day over a six-week uCMS exposure period. The dose of RU486 and timing prior to each experience of stress were based on two previous studies [45,46].

### 4.4. Experimental Design, Stress Model, and Behavioral Assessment

Rats were randomized into four groups (*n* = 10): (i) Control (no stress + vehicle); (ii) RU486 (no stress + RU486 1 mg/kg); (iii) uCMS (stress + vehicle); and (iv) uCMS + RU486 (stress + RU486 1 mg/kg). Each group was subject to behavioral assessments prior to uCMS exposure. Behavioral assessments included the sucrose preference test (SPT), open-field test (OFT), and forced swimming test (FST) and were conducted according to the procedure used in a previous study [47]. These tests were conducted to confirm that there were no differences in behavioral parameters between each group. The six-week uCMS was a slightly modified variation of the procedure previously described by other researchers [47,48,49,50]. Control and RU486 rats were housed together with the uCMS and uCMS + RU486 rats. Briefly, uCMS-exposed rats were housed in separate cages and were subjected to the following stressors for 42 days following a random schedule (refer to Supplementary Table S1): (i) soiled cage for 24 h; (ii) 45° cage tilting for 24 h; (iii) overnight illumination; (iv) water deprivation for 24 h; (v) food deprivation for 24 h; (vi) forced swimming for 5 min; (vii) physical restraint for 2 h; and (viii) 95 dB noise exposure for 2 h. The body weight of each rat was recorded at the beginning of the procedure and then once per week during the stress session. On day 43 (24 h after the last stressor), animal behavior assessments were conducted. Following behavioral assessments, the rats were anesthetized with urethane (40%). Blood was collected immediately through the arteria cruralis and then the rats were sacrificed. The bilateral testes, epididymides, and adrenal glands were removed, weighed, and retained. Per rat, one testis was fixed with paraformaldehyde in preparation for paraffin-embedded sectioning and the other testis was divided into three equal parts for RNA extraction, Western blot analysis, and flow cytometry.

### 4.5. Sperm Concentration and Sperm Motility Assessments

Sperm was collected as previously described [16], with slight modifications. Cauda epididymis was punctured followed by incubation in 400 µL human tubal fluid (HTF, Merck KGaA, Darmstadt, Germany) at 37 °C for 10 min. Next, computer-aided sperm analysis (SCA CASA System; Microptic S.L., Barcelona, Spain) was used for sperm concentration and motility assessment.

### 4.6. Serum Corticosterone and Testosterone Levels

The serum levels of corticosterone and testosterone were determined using a rat corticosterone enzyme-linked immunosorbent assay (ELISA) kit (CUSABIO, Wuhan, China) and a rat testosterone ELISA kit (CUSABIO, Wuhan, China) according to the manufacturers’ instructions [51]. Serum levels of corticosterone and testosterone are expressed as ng/mL.

### 4.7. Tissue Histology and Morphometry

The testes were fixed in paraformaldehyde (4%) for 48 h, embedded in paraffin, and sectioned into 5 μm thick sections. The sections were stained with hematoxylin and eosin (H&E) or hematoxylin alone. A Leica optical microscope (Leica Microsystems, Wetzlar, Germany) was used to assess histology and morphometry. To count the number of different testicular cells in situ, three randomly selected tubules in one hematoxylin-stained section of each animal were analyzed according to a previously described procedure [15]. Briefly, the number of Sertoli cells (located in the testicular basement membrane side with clearly identifiable nucleoli) and germ cells at different stages of maturation that included spermatogonia (located in the testicular basement membrane side with deeply stained nuclei), spermatocytes (larger size and deeply stained nuclei), and spermatids (smaller size and lightly stained nuclei) within the seminiferous tubules were manually counted in 10 animals per group.

### 4.8. Western Blot Analysis

Following the removal of blood vessels, fascia, and adipose tissue, testicular tissue was lysed in RIPA lysis buffer (Beyotime, Shanghai, China) for 30 min on ice. Protein concentration was determined using the BCA assay kit (Beyotime, Shanghai, China). Protein samples were subjected to 12% sodium dodecyl sulfate-polyacrylamide gel electrophoresis (SDS-PAGE), then transferred onto a polyvinylidene fluoride membrane (Millipore, Billerica, MA, USA). The membranes were blocked with 3% BSA for 2 h at room temperature followed by overnight incubation with primary antibodies at 4 °C. The membranes were washed four times with Tris-buffered saline plus Tween (TBST) followed by incubation with horseradish peroxidase (HRP)-conjugated antibodies for 1 h at room temperature. Target proteins were detected using an enhanced chemiluminescence kit (Millipore, Billerica, MA, USA). Primary antibodies included p53 rabbit polyclonal antibody (1:1000; Abcam, Cambridge, GBR), cleaved CASPASE3 rabbit polyclonal antibody (1:1000; Abcam, Cambridge, GBR), BAX rabbit polyclonal antibody (1:1000; Bioss, Beijing, China), BCL-2 rabbit polyclonal antibody (1:1000; Abcam, Cambridge, GBR), CDK4 rabbit monoclonal antibody (1:2000; Abcam, Cambridge, GBR), CDK6 mouse monoclonal antibody (1:2000; Cell Signaling Technology [CST], Danvers, MA, USA), CYCLIN D1 rabbit polyclonal antibody (1:1000; Bioss, Beijing, China), p-RB rabbit polyclonal antibody (1:1000; Bioss, Beijing, China), glucocorticoid receptor (GR) rabbit polyclonal antibody (1:1000; Bioss, Beijing, China), and β-actin rabbit monoclonal antibody (1:1000; CST, Danvers, MA, USA). β-actin was used as a loading control.

### 4.9. Terminal Deoxynucleotidyl Transferase-Mediated dUTP-Biotin Nick End Labeling (TUNEL) Assay

Paraffin-embedded sections were stained with the TUNEL assay using an in situ apoptosis kit (Roche, Mannheim, Germany). All the experimental procedures were conducted according to the kit instructions. Seminiferous tubules with more than six TUNEL-positive cells were considered TUNEL-positive tubules; 100 seminiferous tubules were observed by microscopy according to a previously published method [16].

### 4.10. Cellular DNA Content Determination

Germ cells were isolated by discontinuous Percoll density gradient centrifugation and were then further purified by differentiation of velocity of cells sticking to the wall in vitro. Our method was based on a previously published method with a slight modification [52]. Cells were fixed in ice-cold 70% ethanol and were then stored at 4 °C for 24 h. Subsequently, cells were incubated with propidium iodide (PI) and RNase A for 30 min at 37 °C in the dark. Then, DNA content distribution was analyzed using flow cytometry (AccuriC6, BD Biosciences, San Jose, CA, USA).

### 4.11. Immunofluorescence Staining

Testicular tissue sections were dewaxed four times with xylene for 20 min. Following antigen retrieval by sodium citrate, the sections were incubated with GR rabbit monoclonal antibody (1:400; CST, Danvers, USA) or IgG rabbit monoclonal antibody (1:400; Beyotime, Beijing, China) for 24 h at 4 °C and were then incubated with HRP-conjugated antibodies for 1 h at room temperature. IgG was used as a negative control. Protein expression was detected using a Leica optical microscope (Leica Microsystems, Wetzlar, Germany) under 400× magnification.

### 4.12. Statistical Analysis

All values are presented as mean ± SD. Normality was tested using the Shapiro–Wilk test and homogeneity of variances was tested using the Levene test. Data were analyzed by one-way analysis of variance (ANOVA) with a post hoc multiple comparison test (using Tukey’s test). Differences in weight change over time were evaluated using a repeated-measures two-way ANOVA. A two-sided *p*-value < 0.05 was used to indicate statistical significance. Statistical analyses were performed using GraphPad Prism version 6.01 for Windows (GraphPad Software, La Jolla California USA, www.graphpad.com).

## Supplementary Materials

Supplementary materials can be found at https://www.mdpi.com/1422-0067/20/18/4470/s1.
