# Supplementary material for: Mechanisms of Stress-Induced Spermatogenesis Impairment in Male Rats Following Unpredictable Chronic Mild Stress (uCMS)
Source: Int J Mol Sci. 2019 Sep 10;20(18):4470. doi: 10.3390/ijms20184470 (PMC6770920; doi:10.3390/ijms20184470)
Supplement: Supplementary file 1 [file ijms-20-04470-s001.zip › ijms-569539-supplementary-for proof/Supplementary Figure S1.pdf]

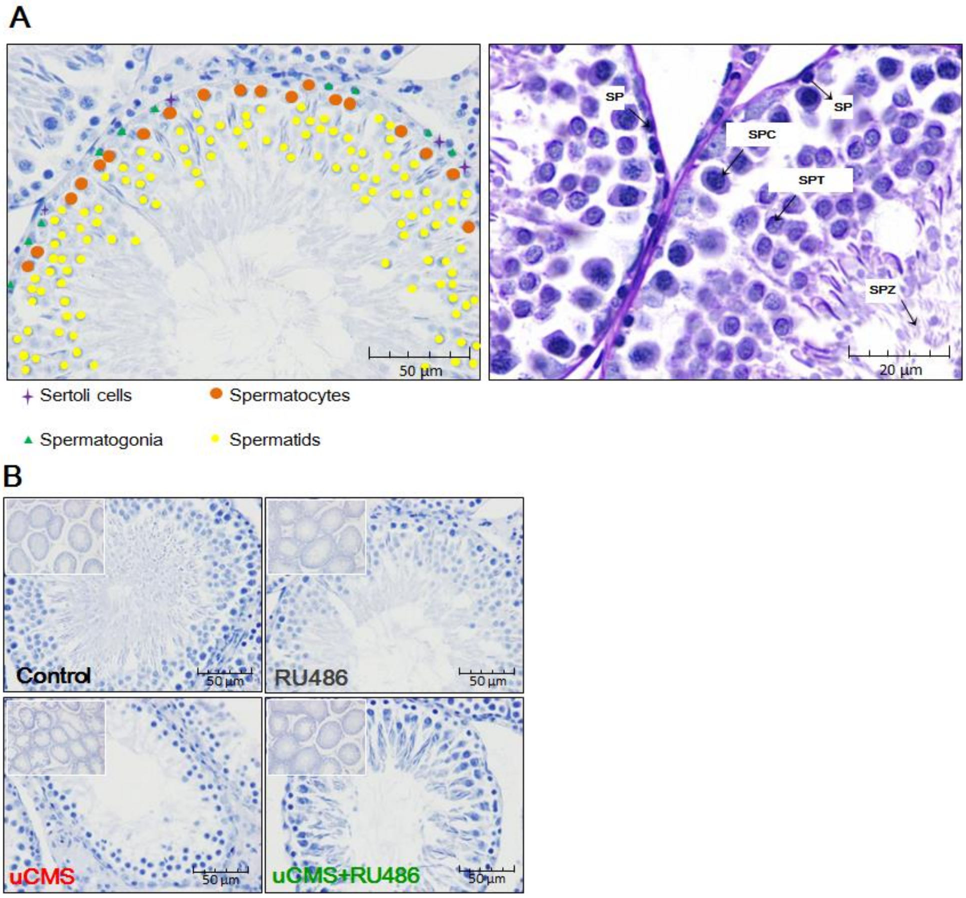

Supplementary Figure S1. Different types of cells within the spermatogenic epithelium. (**A**) A representative image (purple: Sertoli cells; green: spermatogonia; orange: spermatocytes; yellow: spermatids) and a image stained with PAS (Periodic Acid-Schiff) showed the spermatogenic epithelium in an SD rat (400× magnification). Germ cells at different stages of maturation included spermatogonia (SP, located in the testicular basement membrane side with deeply stained nuclei), spermatocytes (SPC, larger size and deeply stained nuclei), and spermatids (SPT, smaller size and lightly stained nuclei). (**B**) Spermatogenic epithelium of SD rats in the control, RU486, uCMS, and uCMS + RU486 groups (400× magnification).
