# Supplementary material for: Mechanisms of Stress-Induced Spermatogenesis Impairment in Male Rats Following Unpredictable Chronic Mild Stress (uCMS)
Source: Int J Mol Sci. 2019 Sep 10;20(18):4470. doi: 10.3390/ijms20184470 (PMC6770920; doi:10.3390/ijms20184470)
Supplement: Supplementary file 1 [file ijms-20-04470-s001.zip › ijms-569539-supplementary-for proof/Supplementary Table S1.pdf]

Supplementary materials.

Supplementary Table S1. The unpredictable chronic mild stress (uCMS) rat model procedure. The above stimulations were arranged for one random type of stimulation per day with no repeat of the same type of stimulation in continuous days, which guarantee that the animal will face unpredictable stimulation. The entire stimulation process lasted for six continuous weeks.

Weeks	Monday	Tuesday	Wednesday	Thursday	Friday	Saturday	Sunday
1th	9:00 body weight			8:00 FST	15:00 OFT(1)	15:00 OFT(2)	9:00 SPT
2th	9:00 body weight， 10:00–next day 10:00 soiled cage	10:00–next day 10:00 deprivation water	10:00–next day 10:00 45°cage tilt	15:00 force swimming for 5 minutes	10:00–18:00 95dB noise	10:00–next day 10:00deprivation food	18:00-next day 8:00overnight illumination
3th	9:00 body weight，10:00–14:00 physical restraint	10:00–18:00 95dB noise	10:00-next day 10:00 deprivation water	10:00–next day 10:00 soiled cage	10:00–14:00 physical restraint	15:00 force swimming for 5 minutes	10:00–next day 10:00 45°cage tilt
4th	9:00 body weight，18:00-next day 8:00overnight illumination	10:00–next day 10:00deprivation food	10:00–14:00 physical restraint	18:00-next day 8:00overnight illumination	15:00 force swimming for 5 minutes	10:00–next day 10:00 deprivation water	10:00–next day 10:00 soiled cage
5th	9:00 body weight，10:00–18:00 95dB noise	10:00–next day 10:00deprivation food	10:00–next day 10:00 45°cage tilt	10:00–14:00 physical restraint	10:00–next day 10:00 deprivation water	15:00 force swimming for 5 minutes	10:00–next day 10:00 soiled cage
6th	9:00 body weight，18:00-next day 8:00overnight illumination	10:00–next day 10:00deprivation food	10:00–next day 10:00 45°cage tilt	10:00–18:00 95dB noise	10:00–next day 10:00 45°cage tilt	15:00 force swimming for 5 minutes	10:00–next day 10:00 deprivation water
7th	9:00 body weight，10:00–18:00 95dB noise	10:00–next day 10:00deprivation food	10:00–next day 10:00 soiled cage	10:00–14:00 physical restraint	18:00-next day 8:00overnight illumination	10:00–next day 10:00 soiled cage	18:00-next day 8:00overnight illumination
